# Physiological and anthropometric determinants of critical power, *W*′ and the reconstitution of *W*′ in trained and untrained male cyclists

**DOI:** 10.1007/s00421-020-04459-6

**Published:** 2020-08-09

**Authors:** Alan Chorley, Richard P. Bott, Simon Marwood, Kevin L. Lamb

**Affiliations:** 1 Department of Sport and Exercise Sciences, University of Chester, Chester, CH1 4BJ UK; 2 School of Health Sciences, Liverpool Hope University, Liverpool, L16 9JD UK

**Keywords:** *W*′ reconstitution, Cycling, Fatigue, Recovery, Correlation

## Abstract

**Purpose:**

This study examined the relationship of physiological and anthropometric characteristics with parameters of the critical power (CP) model, and in particular the reconstitution of *W*′ following successive bouts of maximal exercise, amongst trained and untrained cyclists.

**Methods:**

Twenty male adults (trained nine; untrained 11; age 39 ± 15 year; mass 74.7 ± 8.7 kg; *V̇*O_2max_ 58.0 ± 8.7 mL kg^−1^ min^−1^) completed three incremental ramps (20 W min^−1^) to exhaustion interspersed with 2-min recoveries. Pearson’s correlation coefficients were used to assess relationships for *W*′ reconstitution after the first recovery (*W*′_rec1_), the delta in *W*′ reconstituted between recoveries (∆*W*′_rec_)_,_ CP and *W*′.

**Results:**

CP was strongly related to *V̇*O_2max_ for both trained (*r* = 0.82) and untrained participants (*r* = 0.71), whereas *W*′ was related to *V̇*O_2max_ when both groups were considered together (*r* = 0.54). *W*′_rec1_ was strongly related to *V̇*O_2max_ for the trained (*r* = 0.81) but not untrained (*r* = 0.18); similarly, ∆*W*′_rec_ was strongly related to *V̇*O_2max_ (*r* = − 0.85) and CP (*r* = − 0.71) in the trained group only.

**Conclusions:**

Notable physiological relationships between parameters of aerobic fitness and the measurements of *W*′ reconstitution were observed, which differed among groups. The amount of *W*′ reconstitution and the maintenance of *W*′ reconstitution that occurred with repeated bouts of maximal exercise were found to be related to key measures of aerobic fitness such as CP and *V̇*O_2max_. This data demonstrates that trained cyclists wishing to improve their rate of *W*′ reconstitution following repeated efforts should focus training on improving key aspects of aerobic fitness such as *V̇*O_2max_ and CP.

## Introduction

The two-parameter critical power model formulated by Monod and Scherrer (1965) describes how the hyperbolic relationship between power output and time to exhaustion in the ‘severe’ intensity domain is comprised of the parameters; critical power (CP) and the finite capacity of work available during exercise above CP, known as *W*′ (Jones and Vanhatalo 2017). The severe intensity domain is characterised by an inability to maintain metabolic homeostasis, with consequent rises in oxygen uptake (*V̇*O_2_) until the limit of tolerance ensues (Jones et al. 2019). CP thus marks the boundary between the heavy and severe intensity domains (Jones et al. 2019), below which homeostasis is achieved, albeit with a delayed metabolic steady-state and elevated blood lactate [BLa^−^] relative to baseline values. Known values of CP and *W*′ can be used to predict time to exhaustion in the severe intensity domain (Jones and Vanhatalo 2017). However, in the context of cycling most forms of races from track cycling team pursuits to Grand-Tour stages are characterised by intermittent efforts within the severe intensity domain interspersed with periods below CP (Craig and Norton 2001; Vogt et al. 2007) which allows partial or complete reconstitution of *W*′ depending on the recovery duration (Chidnok et al. 2013a).

*W*′ was originally thought to represent the anaerobic capacity derived from anaerobic glycolysis, phosphocreatine (PCr) and oxygen stores within the muscle (Miura et al. 1999). Latterly, *W*′ has been related to an accumulation of fatiguing metabolites such as adenosine diphosphate, inorganic phosphate and hydrogen ions (Ferguson et al. 2010), a notion supported by a reduction in *W*′ due to impaired dissipation of metabolites following prior exercise in different muscle groups (Johnson et al. 2014). In untrained populations, the magnitude of *W*′ has been linked to muscle glycogen availability (Clark et al. 2019; Miura et al. 2000), the development of the *V̇*O_2_ slow component (Jones and Vanhatalo 2017; Murgatroyd et al. 2011), the depletion of PCr (Chidnok et al. 2013a), leg morphology (Byrd et al. 2017; Miura et al. 2002), though not muscle fibre type distribution (Vanhatalo et al. 2016). In a well-trained population, leg-strength, but not quadriceps volume was correlated with the magnitude of *W*′ (Kordi et al. 2018).

In marked contrast to our understanding of *W*′ depletion, the determinants of the reconstitution kinetics of *W*′ during exercise have received little scientific attention, despite being crucial to performance outcomes dependent upon repeated severe intensity efforts. *W*′ reconstitution kinetics have been demonstrated to be curvilinear with time and most rapid in early recovery (Ferguson et al. 2010) but not consistent with the recovery kinetics of PCr, pH, *V̇*O_2_ or [BLa^−^] (Chidnok et al. 2013b; Ferguson et al. 2010). Subsequently it has been proposed that *W*′ reconstitution kinetics are exponential, with a time constant dependent upon the difference between CP and recovery power output (Skiba et al. 2012, 2015). However, the high degree of individual variability in *W*′ reconstitution kinetics (Bartram et al. 2017; Chorley et al. 2018) suggests there are hitherto unknown multiple contributing factors to *W*′ reconstitution kinetics beyond the difference between CP and recovery power.

Given the dearth of studies examining the potential determinants of *W*′ reconstitution kinetics, the aim of the present study was therefore to assess the relationship between *W*′ reconstitution and physiological characteristics, in trained cyclists and a group of healthy individuals not undertaking regular endurance exercise. Since previous studies have demonstrated aerobic fitness to be associated with improved maintenance of repeated sprint performances (Buchheit and Ufland 2011; McMahon and Wenger 1998; Tomlin and Wenger 2001), which are likely to be at least partly dependent upon *W*′ reconstitution, we hypothesised that markers of aerobic fitness such as CP, *V̇*O_2max_ and training volume would be most closely associated with the capacity to recover *W*′.

## Methods

### Participants

Following institutional ethical approval, twenty adult males (age 39 ± 15 year; body mass 74.7 ± 8.7 kg) volunteered to participate in the study and provided written informed consent. The group comprised two subsets; trained endurance cyclists undertaking a minimum 5 h per week structured cycle training (*n* = 9; age 39 ± 12 year; body mass 72.7 ± 7.1 kg), and untrained (*n* = 11; age 39 ± 18 year; body mass 76.3 ± 9.9 kg) who were engaged in active sport on a minimum weekly basis (team sports or sprinting) and undertook less than 2 h per week of recreational cycling.

### Experimental design

Participants completed three sessions over a period of 5–8 days with a minimum of 2 days between visits. Sessions were completed in an air-conditioned laboratory (temperature 19 ± 1 °C; humidity 54% ± 5%) at similar times of day (± 1.5 h). Participants undertook each session having avoided strenuous exercise and alcohol consumption for 24 h, caffeine for 4 h, and were 3 h post-prandial. The first visit comprised anthropometric and body composition measures, and baseline physiological testing incorporating a ramp all-out test to determine CP and *W*′. The ramp all-out test has been shown to produce valid estimates of CP and *W*′ from a single laboratory visit (Murgatroyd et al. 2014). During visit two, participants completed a familiarisation of the repeated ramp test (RRT) protocol which has been shown to produce reliable measurements of *W*′ reconstitution (Chorley et al. 2018), whilst visit three comprised the same RRT with all measurements being recorded for analysis. Cycle tests were performed on an electronically braked cycle ergometer (Lode Excalibur Sport, Lode BV, Groningen, Netherlands) adjusted for each participant’s comfort and pedal type (standardised for all visits). Pulmonary gas exchange was sampled breath-by-breath with an on-line analyser (Quark CPET, Cosmed, Rome, Italy) calibrated prior to each test with gases of known concentrations and volumes. Participants were fitted with a wireless ANT + chest strap (Garmin International, Kansas) for continuous monitoring of heart rate.

### Procedures

#### Anthropometric assessments

Each participant was measured for stature, body mass, and body composition by air displacement plethysmography (BodPod, Life Measurement Instruments, Concord, CA; calibrated prior to each visit). Skinfold thicknesses were measured to 0.1 mm using Harpenden callipers (British Indicators, Luton, UK) at nine anatomical sites on the right-hand side of the body (triceps; biceps; subscapular; pectoral; supraspinal; iliac crest; abdominal; thigh; calf) in accordance with procedures recommended by the International Society for the Advancement of Kinanthropometry. Thigh and calf girths were measured using an anthropometric tape to 0.1 cm midway between the inguinal crease and the proximal border of the patella and the maximum calf on the right side of the body respectively. Corresponding muscle girths were calculated as Eq. 1:

1 $${\text{Muscle girth }} = {\text{ leg girth }}{-} \, \left( {\pi \, \times {\text{ Sk}}\inf {\text{old}} - {\text{thickness}}} \right)$$

### Baseline physiological testing

**Fig. 1:**
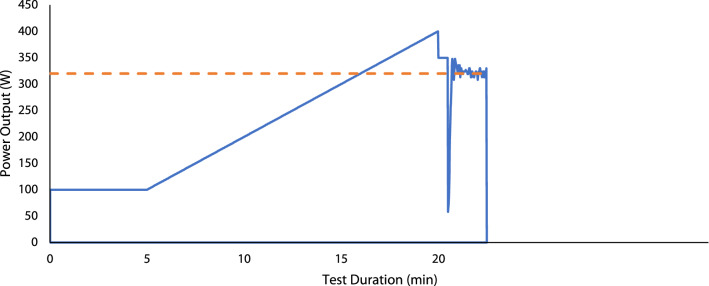
Example baseline test power output profile. Dashed horizontal line indicates critical power

For the accurate determination of CP, the ergometer was configured with a ‘linear factor’ (LF) (Chorley et al. 2018; Murgatroyd et al. 2014) based on a predicted CP and preferred cadence derived from examination of participants’ recent training or race data (where available), otherwise a predicted power output of 3 × body mass (in kg) (van der Vaart et al. 2014) and a cadence of 80 rev·min^−1^ (Murgatroyd et al. 2014) were used to calculate LF. As shown in Fig. 1 participants cycled for 5 min before transitioning to a 20 W min^−1^ ramp to the limit of tolerance with strong verbal encouragement provided. When cadence was observed to fall to 60 rev min^−1^ power output was immediately stepped down before any recovery could take place to 30 W above predicted CP to ensure full depletion of *W*′. When participants could no longer maintain a cadence of 50 rev min^−1^ at the reduced power output the ergometer was immediately switched from hyperbolic mode into linear mode during which they cycled all-out for 2 min. Knowledge of time to completion of this phase was withheld to minimise pacing. CP was calculated as the mean power output during the final 30 s of the all-out phase of the baseline test (Murgatroyd et al. 2014).

### Experimental trial

**Fig. 2:**
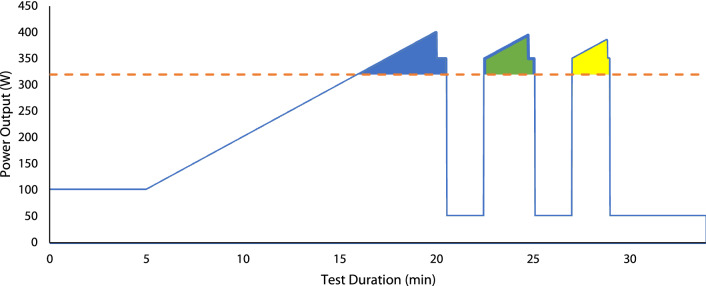
Example repeated ramp test test power output profile. Dashed horizontal line indicates critical power. *W*′ is the area above CP during the initail ramp (first blue shaded area), *W*′ reconstitution occurs when power output falls below CP prior to its expenditure in the subsequent ramp phase. The second (green) shaded area and third (yellow) shaded areas denote *W*′_rec1_ and *W*′_rec2_, respectively

The RRT protocol (see Fig. 2) was performed with the ergometer in hyperbolic mode and commenced with 5 min cycling at 100 W before transitioning into a 20 W min^−1^ ramp to the limit of tolerance. When cadence was observed to fall to 60 rev·min^−1^ power output was immediately stepped down before any recovery could take place to 30 W above CP to ensure full depletion of *W*′ until cadence dropped to 50 rev min^−1^. Following this, a 2-min recovery period at 50 W was provided before completing two further identical ramp and step-down exercise tests, each also separated by a 2-min recovery period at 50 W. Each ramp commenced at CP + 30 W to reduce errors associated with inter-day variability of CP which would otherwise realise additional, but variable, *W*′ reconstitution during the early phase of the ramp below CP (Chorley et al. 2018). The test concluded with a 5 min cool-down at 50 W. Recovery power output was set at 50 W to reflect a minimal load whilst being representative of a true metabolic cost (Boone et al. 2008).

### Data processing

*W*′ was calculated as the work completed above CP during the first ramp and step-down phase during the experimental trial. *W*′ reconstituted during each 2-min recovery period was calculated as the amount of work completed above CP during the subsequent ramp and step-down phase. *W*′_rec1_ and *W*′_rec2_ were defined as *W*′ reconstituted during the first recovery and second recovery periods, respectively; the difference between *W*′ reconstitution during the first and second recovery periods was defined as ∆*W*′_rec_. The *W*′ reconstitution time constant τ was calculated based on an exponential recovery of *W*′ (Vinetti et al. 2017) using the natural logarithm Eq. 2:

2 $$\tau = - 120/{\text{LN}}\left( {1 - W_{rec1}^{\prime} /W^{\prime} } \right)$$

For pulmonary gas exchange parameters, errant breaths, defined where *V̇*O_2_ differed from the local mean by ≥ 4 SD, were removed. Thereafter gas exchange and power data were interpolated and time aligned to produce second-by-second data for the trial using custom spreadsheets. *V̇*O_2max_ for each participant was deemed to be the maximum mean *V̇*O_2_ recorded over a 30-s period across all tests (Day et al. 2003). *V̇*O_2_ at CP was determined from the intersect of CP and the slope of *V̇*O_2_ against power output during the first ramp to exhaustion during the experimental trial. Heart rate (HR) recovery was calculated as the difference in HR at the end of each ramp-step-down phase and after each 60 s of the subsequent recovery period. Excess post-exercise oxygen consumption (EPOC) was the total *V̇*O_2_ following the end of the ramp-step-down phase to each 60-s time point during the subsequent recovery periods whilst continuing to produce a power output of 50 W.

### Statistical analysis

Descriptive statistics (mean ± SD) were calculated for all the dependent variables and normality of their distributions was checked using the Shapiro–Wilk test. Relationships between the primary variables (CP, *W*′, *W*′_rec1_ and ∆*W*′_rec._) were examined with Pearson’s product-moment correlation coefficient and were calculated for the whole group and separately for each subset (trained and untrained). Independent-samples *t* tests were used to compare the characteristics of the subsets. A one-sample *t* test was used to compare ∆*W*′_rec_ to a 0 J *W*′ reconstitution difference. Paired sample *t* tests were used to compare the differences between *W*′_rec1_ and *W*′_rec2_. Unless stated otherwise all values are absolute. Statistical significance was set at *p* < 0.05 throughout, and analysis was performed using SPSS v.23 (IBM Corp., Armonk, NY).

## Results

### *CP and W*′* characteristics and relationships*

Physical and physiological characteristics including CP and *W*′, along with training history for the group and subsets can be seen in Table 1. Strong positive relationships with *V̇*O_2max_ were evident for CP for the whole group (*r* = 0.86) and both the trained subset (*r* = 0.82), and untrained subset (*r* = 0.71), whilst *W*′ exhibited weaker relationships with *V̇*O_2max_ (whole group: *r* = 0.54; trained: *r* = 0.53; untrained *r* = 0.33). The difference in *V̇*O_2max_ and *V̇*O_2_ at CP was related with *W*′ for the whole group (*r* = 0.66) and both subsets (trained: *r* = 0.65; untrained: *r* = 0.75), whilst positive relationships were observed between the difference in *V̇*O_2max_ and *V̇*O_2_ at CP and *τ* (whole group: *r* = 0.41; trained: *r* = 0.17; untrained: *r* = 0.67). Within the trained subset age was inversely related to both CP (*r* = − 0.50), and *W*′ (*r* = − 0.53) whilst body mass was related to CP (*r* = 0.67) in the untrained subset only (yet unrelated to *W*′). Body Composition characteristics (see Table 2) exhibit no significant differences between subsets. Measures of body composition (sum of skinfolds and fat mass) show clear distinctions between the trained and untrained subsets in their relationships with CP and *W*′ (see Table 3). Thigh muscle girth and CP were strongly related in the untrained subset (*r* = 0.74), but not in the trained subset (*r* = 0.36). *W*′ was unrelated to the thigh measurements in either subset. Training volume (only applicable to the trained subset) displayed a very strong relationship with CP (*r* = 0.86). CP was strongly related to EPOC during the first minute of each recovery overall (*r* = 0.85) and for each subset (trained: *r* = 0.84; untrained: *r* = 0.87).

**Table 1:** Characteristics of whole group, cycle trained and untrained

	Age	Training	CP	CP	*W*′	*W*′	*V̇*O_2max_	*V̇*O_2max_
	(year)	(h·week^−1^)	(W)	(W·kg^−1^)	(kJ)	(J·kg^−1^)	(L·min^−1^)	(mL·min^−1^·kg^−1^)
Group (*n* = 20)	39 ± 15	N/a	268 ± 43	3.63 ± 0.68	11.0 ± 4.0	151 ± 69	4.29 ± 0.48	58.0 ± 8.7
Trained (*n* = 9)	39 ± 12	8.2 ± 3.6	301 ± 35*	4.17 ± 0.60*	12.7 ± 3.4	178 ± 56	4.59 ± 0.46*	63.7 ± 8.7*
Untrained (*n* = 11)	39 ± 18	N/a	242 ± 28	3.19 ± 0.35	9.6 ± 4.1	128 ± 59	4.03 ± 0.34	53.4 ± 5.7

Physiological measurements in absolute terms and relative terms

*Significantly different (*p* < 0.01) to the untrained subset

**Table 2:** Body composition and anthropometric measurements of whole group, cycle trained and untrained

	Mass	Stature	Body fat	Fat mass	Sum of 9 Skinfolds	Thigh muscle girth	Calf muscle girth
	(kg)	(cm)	(%)	(kg)	(mm)	(mm)	(mm)
Group (*n* = 20)	74.7 ± 8.7	178.1 ± 6.3	17.5 ± 6.0	13.3 ± 5.4	93.9 ± 30.3	467 ± 38	352 ± 24
Trained (*n* = 9)	72.7 ± 7.1	178.1 ± 7.8	16.1 ± 5.9	11.8 ± 4.7	82.5 ± 29.5	475 ± 43	354 ± 22
Untrained (*n* = 11)	76.3 ± 9.9	178.1 ± 5.2	18.7 ± 6.1	14.4 ± 5.8	103.1 ± 28.8	460 ± 33	350 ± 27

**Table 3:** Correlation coefficients between measures of body composition and physiological measures with CP, *W*′, *W*′_rec1,_ ∆*W*′_rec_ and τ

	Measure	CP (W)	*W*′ (J)	*W*′_rec1_ (J)	∆*W*′_rec_ (J)	τ (s)
Group (*n* = 20)	*V̇*O_2max_ (L·min^−1^)	0.86*	0.54*	0.70*	− 0.18	0.19
	CP (W)		0.34	0.49*	− 0.20	0.01
	*W*′ (J)	0.34		0.77*	0.03	0.78*
	Fat mass (kg)	− 0.09	− 0.55	− 0.56*	− 0.16	− 0.22
	Fat free mass (kg)	0.24	0.03	0.05	− 0.15	− 0.01
	Sum of nine skinfolds (mm)	− 0.37	− 0.47*	− 0.60*	− 0.01	− 0.05
	Thigh muscle girth (mm)	0.51*	− 0.07	− 0.01	0.01	− 0.07
	Calf muscle girth (mm)	0.38	− 0.24	− 0.14	0.11	− 0.19
Trained (*n* = 9)	*V̇*O_2max_ (L·min^−1^)	0.82*	0.53	0.81*	− 0.85*	− 0.26
	CP (W)		0.70*	0.52	− 0.71*	− 0.47
	*W*′ (J)	0.70*		0.71*	− 0.31	0.51
	Fat mass (kg)	− 0.07	− 0.40	− 0.56	0.45	0.14
	Fat free mass (kg)	0.14	− 0.07	0.21	− 0.21	− 0.33
	Sum of nine skinfolds (mm)	− 0.38	− 0.47*	− 0.63	0.45	0.14
	Thigh muscle girth (mm)	0.36	− 0.31	− 0.03	0.17	− 0.37
	Calf muscle girth (mm)	0.22	− 0.33	− 0.07	− 0.08	− 0.34
Untrained (*n* = 11)	*V̇*O_2max_ (L·min^−1^)	0.71*	0.33	0.18	0.21	0.47
	CP (W)		0.04	− 0.19	− 0.04	0.10
	*W*′ (J)	0.04		0.91*	0.19	0.94*
	Fat mass (kg)	− 0.28	− 0.56	− 0.70*	− 0.45	− 0.37
	Fat free mass	0.64*	0.14	− 0.06	0.09	0.21
	Sum of nine skinfolds (mm)	0.02	− 0.33	− 0.44	− 0.26	− 0.10
	Thigh muscle girth (mm)	0.74*	− 0.05	− 0.32	− 0.13	0.13
	Calf muscle girth (mm)	0.66*	− 0.28	− 0.45	0.20	− 0.13

*p < 0.05

### Physiological relationships with *W*′ reconstitution

**Fig. 3:**
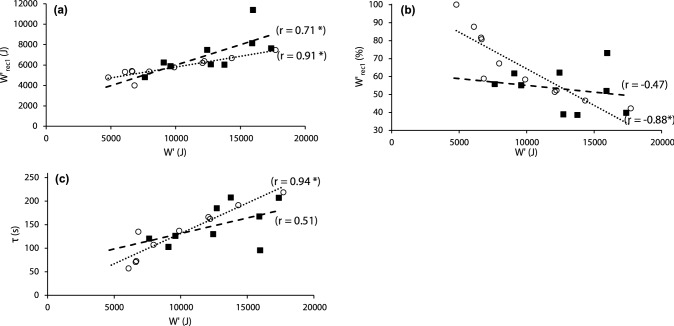
Relationships between *W*′ and the reconstitution of *W*′ during 2-min of recovery. **a** Absolute reconstitution in Joules during the first recovery period. **b** Proportional reconstitution as a percentage of participant’s *W*′ during the first recovery period. **c** The time constant (τ) determined for the participant’s *W*′ during the first recovery period. Trained—Solid black squares and heavy dashed trendline; Untrained—Clear circles and dotted trendline. *p < 0.05

∆*W*′_rec_ was not equal to 0 J for the group or either subset demonstrating a differing of *W*′ reconstitution following successive bouts, paired *t* test comparisons established a significant reduction in *W*′ reconstitution, and thus a slowing of *W*′ reconstitution kinetics, following the second recovery period, versus the first (Table 4). The amount of reconstitution of *W*′ (*W*′_rec1_) was positively correlated with *W*′ for the whole group (*W*′_rec1_: *r* = 0.77), trained (*W*′_rec1_: *r* = 0.71), and untrained subsets (*W*′_rec1_: *r* = 0.91). However, when *W*′_rec1_ was expressed as a proportion of *W*′, the direction of the correlation was reversed (whole group: %*W*′_rec1_: *r* = − 0.78; trained: %*W*′_rec1_: *r* = − 0.47; untrained: %*W*′_rec1_: *r* = − 0.88,). As *τ* is a mathematical derivative of %*W*′_rec1_, τ exhibited similar correlations to *W*′ as shown in Fig. 3. A strong relationship between *W*′_rec1_ and ∆*W*′_rec_ was evident only in the trained subset (*r* = − 0.70).

**Table 4:** *W*′ reconstitution following the first (*W*′_rec1_) and second (*W*′_rec2_) recovery periods of whole group, cycle trained and untrained. Measurements in absolute terms, relative terms and as % of *W*′

	*W*′_rec1_	*W*′_rec1_	% *W*′_rec1_	*W*′_rec2_	*W*′_rec2_	% *W*′_rec2_	∆*W*′_rec_
	(kJ)	(J·kg^−1^)	(%)	(kJ)	(J·kg^−1^)	(%)	(J)
Group (*n* = 20)	6.3 ± 1.6	86.6 ± 26.1	61.9 ± 16.0	5.9 ± 1.8*	81.1 ± 28.4*	58.0 ± 16.7*	398 ± 585**
Trained (*n* = 9)	7.1 ± 1.9	98.8 ± 30.1	56.9 ± 10.5	6.7 ± 2.3*	93.1 ± 35.0*	53.0 ± 12.0*	417 ± 497**
Untrained (*n* = 11)	5.7 ± 0.9	76.6 ± 18.0	66.1 ± 18.8	5.3 ± 0.9*	71.4 ± 18.0*	62.2 ± 19.3*	382 ± 673**

*Significantly different from *W*′_rec1_ (*p* < 0.05)

**Significantly different (*p* < 0.01) from 0 J

CP was positively related to *W*′_rec1_ for the whole group and trained subset (group: *r* = 0.49; trained: *r* = 0.52), and negatively related with ∆*W*′_rec_ for the trained subset only (*r* = − 0.71). Strong relationships between *W*′ reconstitution and *V̇*O_2max_ were evident for the whole group (*W*′_rec1_: *r* = 0.70) and the trained subset (*W*′_rec1_: *r* = 0.81; ∆*W*′_rec_: *r* = − 0.85). The difference in *V̇*O_2max_ and *V̇*O_2_ at CP had similar strong relationships with *W*′ reconstitution for the whole group (*W*′_rec1_: *r* = 0.65) and both subsets (trained: *W*′_rec1_: *r* = 0.66; untrained: *W*′_rec1_: *r* = 0.71), but no relationship with ∆*W*′_rec_.

**Fig. 4:**
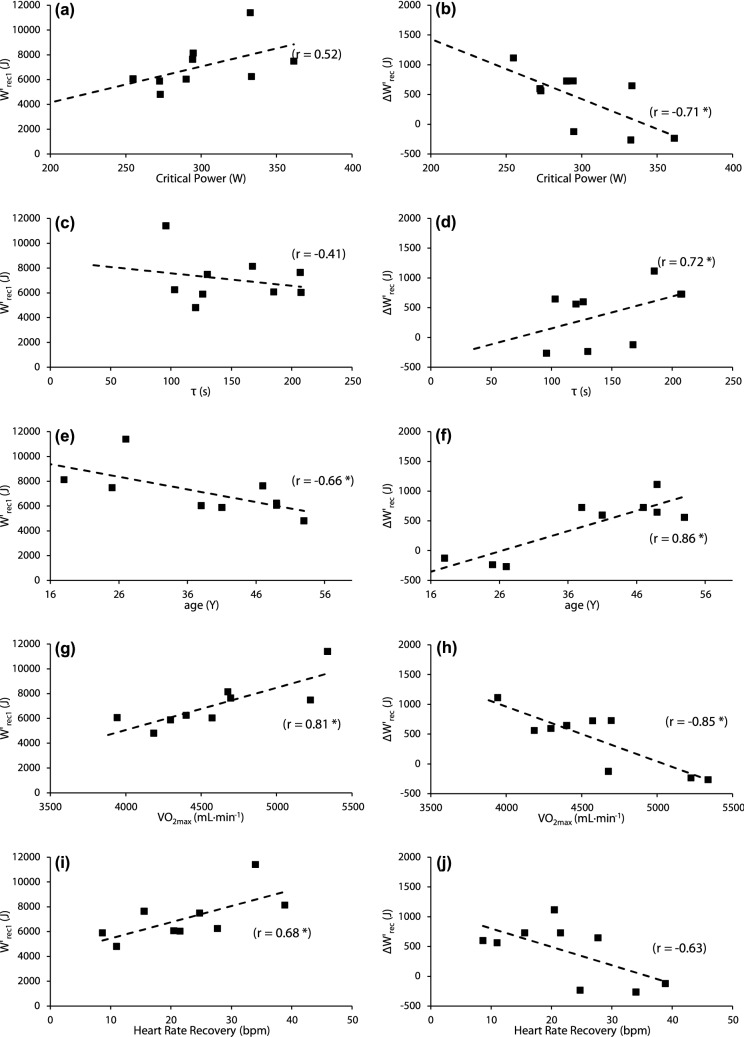
Relationships between factors affecting *W*′ reconstitution in the trained subset. Relationships between critical power and **a** *W*′ reconstitution during the first recovery period, and **b** the difference between *W*′ reconstitution during the first and second recovery periods (∆*W*′_rec_). Relationships between τ and **c** *W*′ reconstitution, and **d** ∆*W*′_rec_. Relationships between age and **e** *W*′ reconstitution, and **f** ∆*W*′_rec_. Relationships between *V̇*O_2max_ and **g** *W*′ reconstitution, and **h** ∆*W*′_rec_. Relationships between heart rate recovery after 60 s of recovery and **i** *W*′ reconstitution, and **j** ∆*W*′_rec_ *p < 0.05

For the group overall (*r* = − 0.43) and the trained subset especially (*r* = − 0.66; Fig. 4e) age was negatively related to *W*′_rec1_, and very strongly related to ∆*W*′_rec_ (*r* = 0.86; Fig. 4f). Training volume (only applicable to the trained subset) displayed a strong negative relationship with ∆*W*′_rec_ (*r* = − 0.75). No significant relationships were observed between parameters of *W*′ reconstitution and age in the untrained subset.

The *W*′ reconstitution relationships with EPOC and HR recovery were strongest during the first minute of each recovery period, particularly for the trained subset, but non-significant among the untrained (see Table 5).

**Table 5:** Correlation coefficients for excess post-oxygen consumption and heart rate recovery during the first minute of each recovery period

		EPOC		HR recovery	
		*W*′_rec1_ (J)	∆*W*′_rec_ (J)	*W*′_rec1_ (J)	∆*W*′_rec_ (J)
Group (*n* = 20)	1st min of 1st recovery	0.40	− 0.08	0.60*	− 0.38
	1st min of 2nd recovery	0.51*	− 0.13	0.56*	− 0.26
	1st min of cool down	0.50*	− 0.14	0.57*	− 0.34
Trained (*n* = 9)	1st min of 1st recovery	0.56	− 0.70*	0.72*	− 0.65*
	1st min of 2nd recovery	0.63*	− 0.74*	0.68*	− 0.63
	1st min of cool down	0.70*	− 0.73*	0.75*	− 0.69*
Untrained (*n* = 11)	1st min of 1st recovery	− 0.21	0.26	0.24	− 0.03
	1st min of 2nd recovery	0.14	0.23	0.19	0.01
	1st min of cool down	− 0.19	− 0.23	0.14	− 0.07

*p < 0.05

### Anthropometric and body composition relationships with *W*′ reconstitution

Body composition (sum of skinfolds and fat mass) exhibited negative relationships with *W*′_rec1_ (see Table 3) for the group and subsets. Neither *W*′_rec1_ nor ∆*W*′_rec_ were related to the thigh or calf girth measurements for the group or either subset.

## Discussion

The principal finding from this investigation was that the capacity for *W*′ reconstitution during a fixed recovery period (*W*′_rec1_) was related to parameters of aerobic fitness in trained cyclists. Moreover, the maintenance of *W*′ reconstitution (∆*W*′_rec_) was also related to aerobic fitness in trained cyclists. Consequently, aerobic fitness appears to be an important determinant of *W*′ reconstitution, a feature not fully accounted for in present models of *W*′ depletion and recovery kinetics. Conversely, despite their good aerobic fitness and similar body composition, such relationships among the active (but not cycle trained) subset were absent. As such, prediction models and investigations of *W*′ reconstitution should account for or control homogeneity of training status within participants.

### *W*′ reconstitution

As might be expected participants with the largest *W*′ had the greatest absolute reconstitution after 2 min of recovery, (Fig. 3a). This is in keeping with previous studies demonstrating an exponential recovery of *W*′ as a function of *W*′ (Skiba et al. 2015; Vinetti et al. 2017). However, when expressed as a percentage of *W*′ recovery, this relationship disappeared in the trained group and was negative in the untrained group, i.e., those with the highest *W*′ reconstituted the smallest fractional of *W*′ during recovery (Fig. 3). This is similarly expressed within the exponential mathematical function describing *W*′ reconstitution kinetics by the positive relationships between τ and *W*′, such a moderating effect of *W*′ on its reconstitution suggests that *W*′ reconstitution kinetics are a factor of both time and *W*′, supporting their inclusion in the *W*′ balance model by Skiba et al. (2012). The present data, however, extend this to indicate independent effects of aerobic fitness on *W*′ reconstitution kinetics (Table 3). Moreover, a reduction in the reconstitution rate of *W*′ with successive bouts of severe intensity exercise, presumably a feature of the fatigue process, is also not accounted for in the *W*′ balance model. However, such a phenomenon is evidenced by ∆*W*′_rec_ being significantly greater than zero. In the trained subset those with the greatest *W*′_rec1_ had the smallest ∆*W*′_rec_ (i.e., had the smallest fatiguing effect on *W*′ reconstitution), which in turn was strongly related to CP and *V̇*O_2max_ and consequently to aerobic fitness (see Table 3). This reduction in the fatiguing effect with higher aerobic fitness is similar to that seen during repeated sprints (Tomlin and Wenger 2001). The influence of aerobic fitness on exercise performance would therefore seem to extend to recovery, a quality not evident in the active but not cycle trained subset in the present study.

The rate of *W*′ reconstitution has been associated with the restoration of PCr (Chidnok et al. 2013b), which in turn appears to be dependent on oxygen availability (Haseler et al. 1999). In the present study EPOC and HR recovery were both strongly related to *W*′_rec1_ and ∆*W*′_rec_, at least in the trained subset (see Table 5). The former may be reflective of performance on the preceding task (i.e., high CP and *V̇*O_2max_) and the latter is also indicative of high aerobic fitness (Darr et al. 1988). It is possible therefore that the effects of high aerobic fitness extends to improved availability of oxygen during the recovery process. Indeed, as seen in Fig. 4j those with the largest absolute difference in HR following one minute of recovery maintained their *W*′ reconstitution in the final recovery period (i.e., reduced ∆*W*′_rec_).

A novel outcome of this investigation was the observation of inverse relationships shown in Fig. 4e, f between age and *W*′ reconstitution, suggesting that for trained cyclists, advancing age both slows reconstitution and, in particular, exacerbates the slowing of the rate of *W*′ reconstitution that occurs following repeated maximal exercise. This effect may have been confounded by reductions in training volume and thus aerobic fitness with ageing. Indeed, decreases in endurance performance with ageing can be partly mitigated by maintaining training volume (Reaburn and Dascombe 2008). Training volume was inversely related to ∆*W*′_rec_, though not with *W*′_rec1_, in the present study and there were inverse correlations between age and CP (*r* = − 0.49) and training volume (*r* = − 0.61). Nevertheless, the strength of the relationship between age and ∆*W*′_rec_ indicates that ageing may exert an independent effect on the capacity to reconstitute *W*′ following recovery from repeated sever intensity bouts of exercise.

The difference in *V̇*O_2_ at CP and *V̇*O_2max_ represents exercise intensities above CP which elicit the development of the *V̇*O_2_ slow component until *V̇*O_2max_ is attained (Murgatroyd et al. 2011), the extent of which is also associated with the magnitude of *W*′ (Simpson et al. 2015). Similarly, the present study extends that relationship to absolute *W*′ reconstitution but, found no association with the maintenance of *W*′ reconstitution. That τ was only related to the difference in *V̇*O_2_ at CP and *V̇*O_2max_ in the untrained subset may be explained by the multi-faceted aspects of aerobic fitness in the trained group and their relatively high proportion of *V̇*O_2max_ at CP.

For the whole group and both subsets, body mass was unrelated to *W*′ reconstitution or ∆*W*′_rec_, thus providing no justification for normalising these measures per kilogram in the present study as has been suggested recently (Kordi et al. 2018). Whilst body mass itself was unrelated, body composition in terms of fat mass (but not lean mass) and sum of skinfolds was inversely related to *W*′ reconstitution without affecting the ∆*W*′_rec_. Blood flow to adipose tissue adjacent to exercising muscle has been shown to increase during exercise (Heinonen et al. 2012), raising the possibility that competing demands for blood flow could prove detrimental to *W*′ reconstitution, however further research is warranted to confirm this.

### CP and *W*′

As indicated above, increasing age appeared to have a deleterious effect on some performance measures, particularly among the trained subset. The effects of age on the decline in both *V̇*O_2max_ and lactate threshold in the trained population are well established (Tanaka and Seals 2008), reflecting the decline in CP observed in the present study. Maintaining training volume and intensity can go some way to mitigate the aerobic decline with advancing age (Tanaka and Seals 2008) and as the present study suggests, training volume itself is strongly associated with CP.

Body mass has been shown to be a determinant of CP in untrained participants (van der Vaart et al. 2014), and whilst the current study reinforces this assertion in a non-cycle trained active population, the relationship did not extend to the cycle trained participants. Whilst body mass itself was unrelated to *W*′, both body composition in terms of fat mass (but not lean mass) and sum of skinfolds was negatively associated with *W*′. Interestingly, these body composition relationships were consistent across the whole group and subsets indicating that the connections between *W*′ and fat mass are independent of cycle-specific training. In contrast to previous studies (Byrd et al. 2017; Miura et al. 2002), thigh anthropometrics showed no associations with *W*′ or its reconstitution. Yet, unlike the previous studies few of the current participants undertook regular strength training. It has been shown that cross sectional area of type I muscle fibres correlates with CP in endurance athletes (Mitchell et al. 2018) supporting the anthropometric correlates in the present study, and indicating that hypertrophy of different fibre types due to strength or endurance training is likely to be a determinant of *W*′ and CP respectively.

## Conclusions

The novel finding of the present study was that in trained cyclists, the rate of reconstitution of *W*′, and in particular the reduction in *W*′ reconstitution that occurred with repeated bouts of severe intensity exercise, were correlated with parameters of aerobic fitness, such as CP and *V̇*O_2max_. Furthermore, whilst all the current participants undertook regular exercise, there were inconsistencies between the trained cyclists and the recreationally active (untrained) group with respect to the relationship between aerobic fitness and parameters of *W*′ reconstitution. The present data therefore also reiterates the need to use trained participants in studies that allude to performance and its underlying physiological determinants. Hence, mathematical models developed to predict the reconstitution of *W*′ in trained cyclists involving repeated severe intensity bouts of exercise should account for aerobic fitness per se.

In examining the determinants of *W*′ reconstitution, the present study provides athletes and coaches with an insight into the factors which can influence performance in events dependent upon repeated severe intensity exercise, including many track and road cycling events. Consequently, the development of aerobic fitness (e.g., *V̇*O_2max_ and CP) appears to be important in maximising the rate of *W*′ reconstitution and minimising the decrement in the rate of reconstitution that occurs during intermittent severe intensity exercise.

## Abbreviations

BLa^−^: Blood lactate
CP: Critical power
EPOC: Excess post-exercise oxygen consumption
HR: Heart rate
LF: Linear factor
PCr: Phosphocreatine
RRT: Repeated ramp test
*V̇*O_2_: Oxygen uptake
*V̇*O_2max_: Maximum oxygen uptake
*W*′: The finite capacity of work above critical power
*W*′_rec1_: Amount of *W*′ reconstituted during first recovery period
*W*′_rec2_: Amount of *W*′ reconstituted during second recovery period
∆*W*′_rec_: Delta of *W*′ reconstituted between first and second recovery periods
*τ*: Tau, the time constant of *W*′_rec1_

## References

[CR1] Bartram JC, Thewlis D, Martin DT, Norton KI (2017). Accuracy of *W*′ recovery kinetics in high performance cyclists—modelling intermittent work capacity. Int J Sports Physiol Perform.

[CR2] Boone J, Koppo K, Bouckaert J (2008). The VO2 response to submaximal ramp cycle exercise: influence of ramp slope and training status. Resp Physiol Neurobi.

[CR3] Buchheit M, Ufland P (2011). Effect of endurance training on performance and muscle reoxygenation rate during repeated-sprint running. Eur J Appl Physiol.

[CR4] Byrd MT, Switalla JR, Eastman JE, Wallace BJ, Clasey JL, Bergstrom HC (2017). Contributions of body composition characteristics to critical power and anaerobic work capacity. Int J Sports Physiol Perform.

[CR5] Chidnok W, DiMenna FJ, Fulford J, Bailey SJ, Skiba PF, Vanhatalo A, Jones AM (2013). Muscle metabolic responses during high-intensity intermittent exercise measured by (31)P-MRS: relationship to the critical power concept. Am J Physiol Regul Integr Comp Physiol.

[CR6] Chidnok W, Fulford J, Bailey SJ, Dimenna FJ, Skiba PF, Vanhatalo A, Jones AM (2013). Muscle metabolic determinants of exercise tolerance following exhaustion: relationship to the “critical power”. J Appl Physiol.

[CR7] Chorley A, Bott RP, Marwood S, Lamb KL (2018). Reconstitution of *W*′ in recovery slows with repeated bouts of maximal exercise. Int J Sports Physiol Perform.

[CR8] Clark IE, Vanhatalo A, Thompson C, Joseph C, Black MI, Blackwell JR, Wylie LJ, Tan R, Bailey SJ, Wilkins BW, Kirby BS, Jones AM (2019). Dynamics of the power-duration relationship during prolonged endurance exercise and influence of carbohydrate ingestion. J Appl Physiol.

[CR9] Craig NP, Norton KI (2001). Characteristics of track cycling. Sports Med.

[CR10] Darr KC, Bassett DR, Morgan BJ, Thomas DP (1988). Effects of age and training status on heart rate recovery after peak exercise. Am J Physiol.

[CR11] Day JR, Rossiter HB, Coats EM, Skasick A, Whipp BJ (2003). The maximally attainable Vo(2) during exercise in humans: the peak vs. maximum issue. J Appl Physiol.

[CR12] Ferguson C, Rossiter HB, Whipp BJ, Cathcart AJ, Murgatroyd SR, Ward SA (2010). Effect of recovery duration from prior exhaustive exercise on the parameters of the power-duration relationship. J Appl Physiol.

[CR13] Haseler LJ, Hogan MC, Richardson RS (1999). Skeletal muscle phosphocreatine recovery in exercise-trained humans is dependent on O_2_ availability. J Appl Physiol.

[CR14] Heinonen I, Bucci M, Kemppainen J, Knuuti J, Nuutila P, Boushel R, Kalliokoski KK (2012). Regulation of subcutaneous adipose tissue blood flow during exercise in humans. J Appl Physiol.

[CR15] Johnson MA, Mills DE, Brown PI, Sharpe GR (2014). Prior upper body exercise reduces cycling work capacity but not critical power. Med Sci Sports Exerc.

[CR16] Jones AM, Vanhatalo A (2017). The ‘critical power’ concept: applications to sports performance with a focus on intermittent high-intensity exercise. Sports Med.

[CR17] Jones AM, Burnley M, Black MI, Poole DC, Vanhatalo A (2019). The maximal metabolic steady state: redefining the ‘gold standard’. Physiol Rep.

[CR18] Kordi M, Menzies C, Parker Simpson L (2018). Relationship between power–duration parameters and mechanical and anthropometric properties of the thigh in elite cyclists. Eur J Appl Physiol.

[CR19] McMahon S, Wenger HA (1998). The relationship between aerobic fitness and both power output and subsequent recovery during maximal intermittent exercise. J Sci Med Sport.

[CR20] Mitchell EA, Martin NRW, Bailey SJ, Ferguson RA (2018). Critical power is positively related to skeletal muscle capillarity and type I muscle fibers in endurance-trained individuals. J Appl Physiol.

[CR21] Miura A, Kino F, Kajitani S, Sato H, Sato H, Fukuba Y (1999). The effect of oral creatine supplementation on the curvature constant parameter of the power-duration curve for cycle ergometry in humans. Jpn J Physiol.

[CR22] Miura A, Sato H, Sato H, Whipp BJ, Fukuba Y (2000). The effect of glycogen depletion on the curvature constant parameter of the power-duration curve for cycle ergometry. Ergonomics.

[CR23] Miura A, Endo M, Sato H, Sato H, Barstow TJ, Fukuba Y (2002). Relationship between the curvature constant parameter of the power-duration curve and muscle cross-sectional area of the thigh for cycle ergometry in humans. Eur J Appl Physiol.

[CR24] Monod H, Scherrer J (1965). The work capacity of a synergic muscular group. Ergonomics.

[CR25] Murgatroyd SR, Ferguson C, Ward SA, Whipp BJ, Rossiter HB (2011). Pulmonary O-2 uptake kinetics as a determinant of high-intensity exercise tolerance in humans. J Appl Physiol.

[CR26] Murgatroyd SR, Wylde LA, Cannon DT, Ward SA, Rossiter HB (2014). A ‘ramp-sprint’ protocol to characterise indices of aerobic function and exercise intensity domains in a single laboratory test. Eur J Appl Physiol.

[CR27] Reaburn P, Dascombe B (2008). Endurance performance in masters athletes. Eur Rev Aging Phys Act.

[CR28] Simpson LP, Jones AM, Skiba PF, Vanhatalo A, Wilkerson D (2015). Influence of hypoxia on the power-duration relationship during high-intensity exercise. Int J Sports Med.

[CR29] Skiba PF, Chidnok W, Vanhatalo A, Jones AM (2012). Modeling the expenditure and reconstitution of work capacity above critical power. Med Sci Sports Exerc.

[CR30] Skiba PF, Fulford J, Clarke DC, Vanhatalo A, Jones AM (2015). Intramuscular determinants of the ability to recover work capacity above critical power. Eur J Appl Physiol.

[CR31] Tanaka H, Seals DR (2008). Endurance exercise performance in Masters athletes: age-associated changes and underlying physiological mechanisms. J Physiol-London.

[CR32] Tomlin DL, Wenger HA (2001). The relationship between aerobic fitness and recovery from high intensity intermittent exercise. Sports Med.

[CR33] van der Vaart H, Murgatroyd SR, Rossiter HB, Chen C, Casaburi R, Porszasz J (2014). Selecting constant work rates for endurance testing in COPD: the role of the power-duration relationship. Copd.

[CR34] Vanhatalo A, Black M, DiMenna FJ, Blackwell JR, Schmidt JF, Thompson C, Wylie LJ, Mohr M, Bangsbo J, Krustrup P, Jones AM (2016). The mechanistic bases of the power-time relationship: muscle metabolic responses and relationships to muscle fibre type. J Physiol.

[CR35] Vinetti G, Fagoni N, Taboni A, Camelio S, di Prampero PE, Ferretti G (2017). Effects of recovery interval duration on the parameters of the critical power model for incremental exercise. Eur J Appl Physiol.

[CR36] Vogt S, Schumacher YO, Blum A, Roecker K, Dickhuth HH, Schmid A, Heinrich L (2007). Cycling power output produced during flat and mountain stages in the Giro d’Italia: a case study. J Sports Sci.

